# A Fishery Water Quality Monitoring and Prediction Evaluation System for Floating UAV Based on Time Series

**DOI:** 10.3390/s21134451

**Published:** 2021-06-29

**Authors:** Lei Cheng, Xiyue Tan, Dong Yao, Wenxia Xu, Huaiyu Wu, Yang Chen

**Affiliations:** 1 School of Information Science and Engineering, Wuhan University of Science and Technology, Wuhan 430205, China; chenglei@wust.edu.cn (L.C.); insteind@gmail.com (D.Y.); wuhy@wust.edu.cn (H.W.); chenyag@wust.edu.cn (Y.C.); 2 Hubei Key Laboratory of Intelligent Robot, Wuhan Institute of Technology, Wuhan 430205, China; xuwenxia@wit.edu.cn

**Keywords:** water quality monitoring, six-rotor floating UAV, time series analysis, prediction, evaluation

## Abstract

In recent years, fishery has developed rapidly. For the vital interests of the majority of fishermen, this paper makes full use of Internet of Things and air–water amphibious UAV technology to provide an integrated system that can meet the requirements of fishery water quality monitoring and prediction evaluation. To monitor target water quality in real time, the water quality monitoring of the system is mainly completed by a six-rotor floating UAV that carries water quality sensors. The GPRS module is then used to realize remote data transmission. The prediction of water quality transmission data is mainly realized by the algorithm of time series comprehensive analysis. The evaluation rules are determined according to the water quality evaluation standards to evaluate the predicted water quality data. Finally, the feasibility of the system is proved through experiments. The results show that the system can effectively evaluate fishery water quality under different weather conditions. The prediction accuracy of the pH, dissolved oxygen content, and ammonia nitrogen content of fishery water quality can reach 99%, 98%, and 99% on sunny days, and reach 92%, 98%, and 91% on rainy days.

## 1. Introduction

According to the official website of the US Bureau of Labor Statistics and the China Fishery Ship Safety Analysis Report (1994–2015) statistics, since 2012, the global fishery worker mortality rate has been hovering around 0.08%, which is nearly 59 times that of industrial, mining, and commercial enterprises. The complex soil environment around the waters is one of the main reasons for the tragedy. As the phenomenon of water pollution becomes increasingly serious, various countries give increasingly more attention to fishery safety, and the requirements for water quality are greater than ever. Accordingly, the demand for water quality monitoring devices is also increasing.

On 10 July 2017, the Food and Agriculture Organization of the United Nations (FAO) and the Organization for Economic Cooperation and Development (OECD) jointly released the Agriculture Outlook 2017–2026 report. The report made preliminary predictions on the development of global fisheries in the next ten years. FAO predicted that by 2026, global fishery production will reach 194 million tons. In the next ten years, global fishery output will continue to grow, but the growth rate will slow significantly. In some underdeveloped areas, the lack of transportation infrastructure, imperfect supply system, and backward sanitation conditions may not be improved in the short term. These restrictive conditions will lead to a decline in the growth rate of fisheries in the next ten years. Therefore, the fishermen have accurate and reliable aquaculture data to improve the yield and quality of fishery products, which will surely become the direction for the future development of fisheries.

The key to establishing a water quality prediction and evaluation system is that it relates to the direct interests of every fisherman. Direct detection of water quality is easily affected by many aspects, and is not time-sensitive and scientific. Once a water quality disaster breaks out, it will bring irreparable losses to fishermen. Therefore, the quality of fishery water needs to be monitored in a scientific way. Real-time prediction and evaluation of factors affecting water quality changes have great practical value and economic benefits. In order to effectively prevent and control water quality emergencies and minimize possible losses, this paper describes a system design. Through the combination of UAV and the Internet of Things, the concept of a smart fishery will be promoted to the majority of fishermen. It realizes early detection, early reporting, and early disposal, which not only provide convenience to fisherman, but also ensures their safety to a certain extent.

### 1.1. Research Based on the UAVs

**Figure 1:**
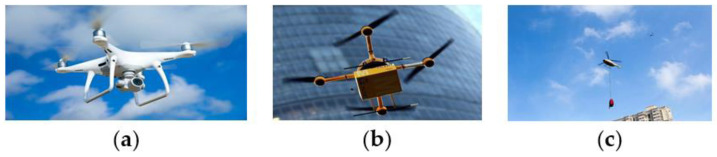
The three major types of UAVs that are experiencing expanded use in the UAVs industry: (**a**) consumer UAVs, (**b**) commercial UAVs, and (**c**) government UAVs.

UAVs [1,2,3] (Figure 1) were first designed to replace humans to complete high-intensity and high-risk work to improve work efficiency. They are developing very rapidly in various fields. Now they have fully penetrated into human life, and have also been fully applied in the field of environmental monitoring. The existing environmental monitoring UAVs [4] are mainly used in two ways [5]. One is to carry a video camera to investigate the area with water pollution and the distribution of garbage. The other is equipped with ambient air monitoring equipment to monitor the concentration of gas or particulate matter in the air or chimney exhaust emissions. Water quality monitoring requires global observations and a large number of local observations in the target area. To obtain a large amount of high-quality local observation information, a UAV system with flexible operation and high controllability is required. It can effectively solve the problem of water environment monitoring and provide powerful help for water quality monitoring. However, in the existing technology, water quality monitoring UAVs have not been fully developed and applied. The main reason is that the system is susceptible to environmental influences such as wind and waves when working near a free-surface. Moreover, the system stability and operation accuracy are low, and the autonomous cruise capability is poor.

**Figure 2:**
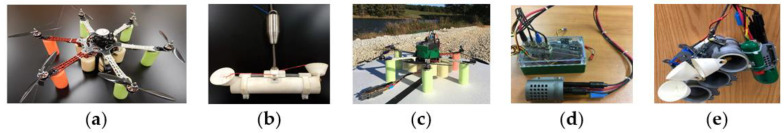
From 2016 to 2020, Koparan et al designed and improved an UAV and its accessories for water quality detection: (**a**) water quality sampling UAV, (**b**) water intake device, (**c**) improved UAV, (**d**) improved sensor array, and (**e**) customized sensor.

It is relatively rare to adopt a water quality sensor array for water quality analysis and monitoring. Ore et al. [6] designed a water quality sampler based on UAV in 2015. The fuselage was equipped with a device that can pump water. The water quality samples were obtained by flying at a fixed height of 1 m above the surface of the water. Koparan et al. designed a water quality sampling UAV (Figure 2a) in 2016. The UAV used a suspended sampler design (Figure 2b). In 2018, he upgraded the water inlet device accordingly [7] (Figure 2c). The sensor (Figure 2d) was replaced for water quality detection, and parameter analysis of water quality status was added [8]. In 2020, he customized the sensor [9] (Figure 2e) and realized the UAV design to monitor the water quality on the surface. The disadvantage was that the data collection in his design was stored in the local hardware, without real-time monitoring. Moreover, due to the limitation of the wheelbase of the model, the UAV had a certain lack of stability, endurance, and load, and could not achieve long-term cross-regional monitoring tasks.

As a kind of aircraft with special performance, multirotor UAVs [10,11] have been recognized and accepted by increasingly more people and enterprises. Compared with traditional fixed wing and helicopter, multirotor UAVs have simple operation, high reliability, good modular performance, high degree of automation, and low gyro benefit, which is the carrier object of this paper.

### 1.2. Background of Water Quality Prediction and Evaluation

The methods of water quality prediction worldwide [12,13,14,15] can be divided into mechanism methods and nonmechanical methods (Table 1). In the actual water quality prediction work, the influencing factors of water quality prediction are very complicated, and the water quality changes do not necessarily nor approximately conform to an exponential distribution. Therefore, the gray model may be powerless, while the time series method can solve these problems. For water quality evaluation, after obtaining certain data, it is classified according to different water quality evaluation standards and stages (Table 2).

To this end, the paper combines the abovementioned outstanding problems to research and designs a full-system solution. The system takes the actual fishery production practice as the starting point, applies the UAV platform as the carrier, and measures common water quality indicators as the research object, and conducts in-depth investigation and analysis of water quality.

## 2. Materials and Methods

This part first conducts an in-depth study on the UAV platform selected to implement the water quality monitoring system. The principles of three electrochemical water quality sensors are then introduced in detail, and the software and hardware of the corresponding platform are designed. Afterward, with reference to national water quality and fishery water quality standards, a quantitative rule sheet for water quality evaluation is developed. Finally, the theoretical method of water quality data prediction based on time series analysis is described.

### 2.1. UAV Floating Structure

This section discusses the aerodynamic characteristics of UAVs in detail. The UAV that can carry an array of water quality monitoring sensors is then selected. Finally, a stable and reliable floating structure is designed, which can meet the smooth takeoff and landing of the UAV on the water, together with the force analyzed by simulation.

#### 2.1.1. Rotor Aerodynamics Analysis

**Figure 3:**
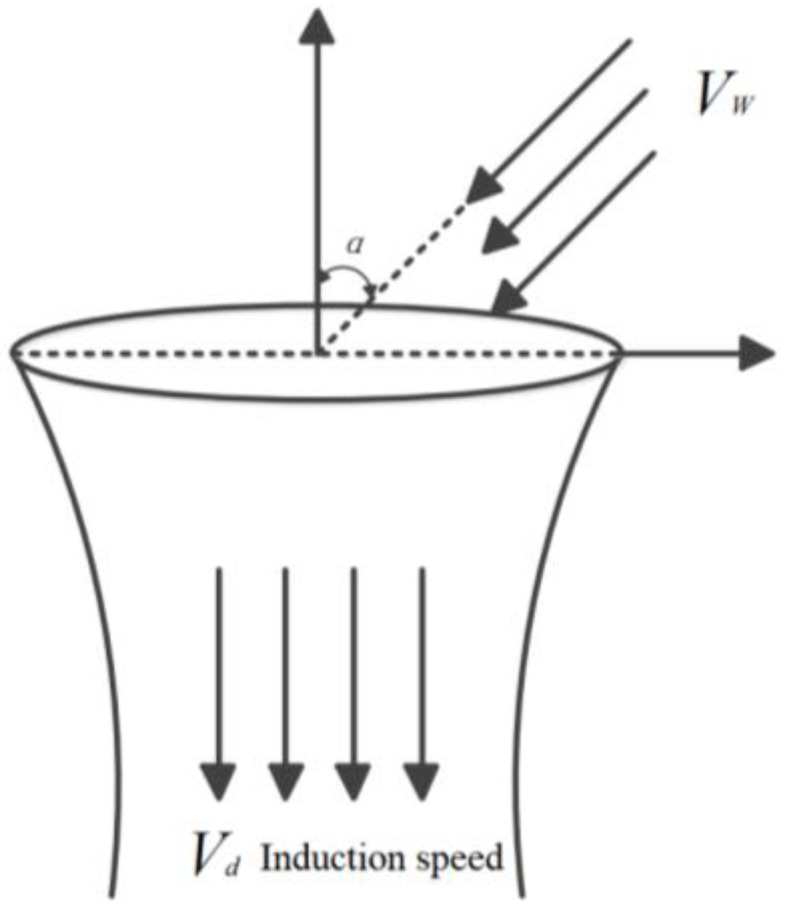
Schematic diagram of the propeller slipstream flow field.

In the process of studying rotor dynamics, the main theoretical basis are blade-element theory [32,33] and momentum theorems [34]. During the rotation of the rotor, there is a pressure difference above and below the plane of rotation. Therefore, the airflow occurs from above the plane of the propeller, and accelerates vertically downward from below to form a very obvious slipstream area. In addition, the air state outside the slipstream area is approximately symmetrical about the rotation axis of the multirotor blades. The air outside the slipstream area corresponds to a static state. The scope of the slipstream area under the entire propeller is relative to the diameter of the propeller disk, which is equivalent to the contracted state. The slipstream flow field of the propeller during rotation is shown in Figure 3.

When rotating, the rotor receives the lift of the rotor. The lift of the rotor is perpendicular to the centerline of the propeller, which can be decomposed into rotor tension and rotor drag. The expressions of rotor tension and drag follow:

(1) $$T_{i}=\frac{1}{2}\rho AC_{T}R^{2}\Omega_{i}^{2}$$

(2) $$M_{i}=\frac{1}{2}\rho AC_{M}R^{2}\Omega_{i}^{2}$$

where ρ is the air density, A is the rotation area of the rotor, $C_{T}$ is the wing dynamic pull coefficient, $C_{M}$ is the dynamic drag coefficient, R is the rotor radius, and $\Omega_{i}$ is the rotation speed of each rotor. The corresponding formulas for $C_{T}$ and $C_{M}$ follow:

(3) $$\frac{C_{T}}{2\sigma a}=\left(\frac{1}{6}+\frac{1}{4}\mu^{2}\right)\theta -\frac{1}{4}\lambda$$

(4) $$\frac{C_{M}}{2\sigma a}=\frac{1}{8\alpha }(1+\mu^{2})\bar{C_{d}}+\left(\frac{1}{6}\theta -\frac{1}{4}\lambda \right)$$

(5) $$\frac{C_{M}}{2\sigma a}=\frac{1}{8\alpha }(1+\mu^{2})\bar{C_{d}}+\left(\frac{1}{6}\theta -\frac{1}{4}\lambda \right)$$

where σ is the solid degree of the rotor, a is the average lift slope value (approximately 5.7), θ is the blade set angle, and μ is the advance ratio, which is defined as

(6) $$\mu =\frac{V_{W}\sin\alpha }{\Omega R}$$

and λ is the inflow ratio, which is defined as

(7) $$\mu =\frac{v_{d}+V_{W}\sin\alpha }{\Omega R}$$

(8) $$v_{d}=\sqrt{-\frac{V_{W}^{2}}{2}+\sqrt{{\left(\frac{V_{W}^{2}}{2}\right)}^{2}+{\left(\frac{T}{2\rho A}\right)}^{2}}}$$

$\bar{C_{{}_{d}}}$ is the average value of backward force of blade element, and $C_{d}$ is the backward force coefficient of blade element, which is defined as follows:

(9) $$C_{d}=\frac{0.144}{RN^{1/5}}$$

In the expression, RN is the Reynolds number; therefore, Formulas (1) and (2) can be simplified as follows:

(10) $$T_{i}=K_{T}\Omega_{i}^{2}$$

(11) $$M_{i}=K_{M}\Omega_{i}^{2}$$

In addition, when the aircraft is hovering, that is, the relative airflow velocity is 0, Formulas (6)–(8) can be simplified as follows:

(12) $$\mu =\frac{V_{M}\sin\alpha }{\Omega R}=0$$

(13) $$\lambda =\frac{v_{d}+V_{M}\sin\alpha }{\Omega R}=\frac{v_{d}}{\Omega R}$$

(14) $$v_{d}=\sqrt{\frac{T}{2\rho A}}$$

#### 2.1.2. Floating Structure Modeling

**Figure 4:**
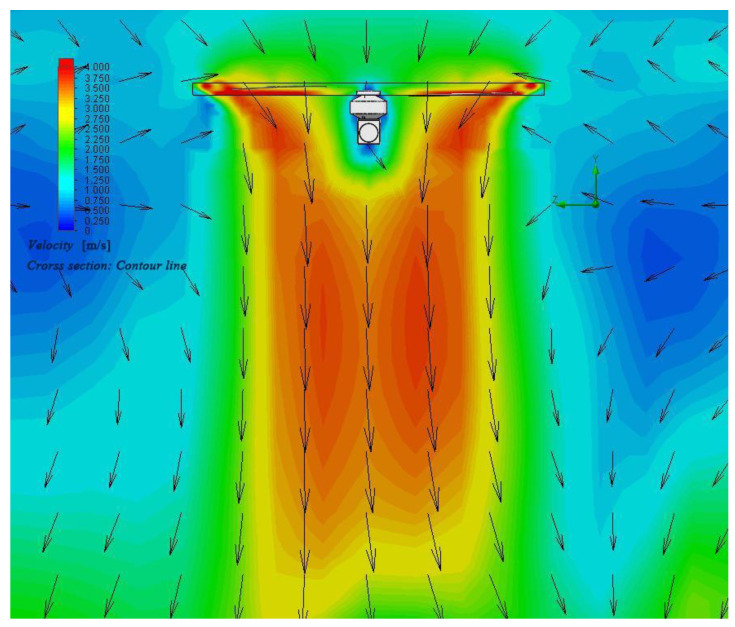
The approximate simulation diagram of the slipstream flow field profile of a single rotor in hovering state.

Based on the rotor aerodynamic analysis [35], a simple ANSYS simulation of the air flow profile of a single rotor is carried out. Finally, the approximate simulation diagram of the slipstream flow field profile of a single rotor in hovering state is shown in Figure 4.

**Figure 5:**
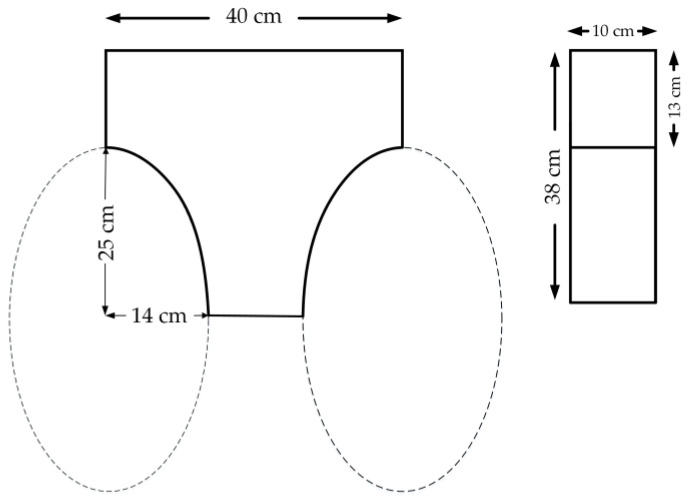
The front and side views of a single rotor.

The intensity of the color in the figure represents the wind speed. The red area has the fastest speed, and the blue area has the slowest speed, which can be regarded as static. Taking the yellow area as the edge of the slipstream, the profile simulation diagram is basically the same as Figure 4. Therefore, based on the slipstream profile, in order to reduce the lateral wind disturbance, the profile is appropriately reduced. The final design takes on a T-like shape, with the front and side views of a single rotor as shown in Figure 5.

In order to weaken the impact of the system hardware on the UAV and the simplicity of the device, the six-rotor was finally selected as the floating water quality monitoring platform.

#### 2.1.3. Floating Structure Simulation Analysis

**Figure 6:**
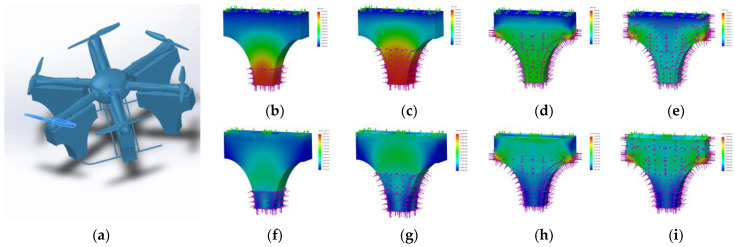
Simulation analysis diagram of the floating structure. (**a**) 3D modeling drawing in SolidWorks; (**b**) deformation simulation at 10 cm immersion depth; (**c**) deformation simulation at 20 cm immersion depth; (**d**) deformation simulation at 30 cm immersion depth; (**e**) deformation simulation at 38 cm immersion depth; (**f**) pressure simulation at 10 cm immersion depth; (**g**) pressure simulation at 20 cm immersion depth; (**h**) pressure simulation at 30 cm immersion depth; and (**i**) pressure simulation at 38 cm immersion depth.

The T-like floating structure of this six-rotor UAV is made of ethylene-vinyl acetate copolymer (EVA) material with the molecular formula $(C_{2}H_{4})x.(C_{4}H_{6}O_{2})y$. After foaming, the foam is produced, which has the characteristics of closed cell structure, nonabsorbent, good water resistance, corrosion resistance, high resilience, and high tension resistance. The proportional 3D modeling diagram in Solidworks is shown in Figure 6a. In this paper, the static analysis of the T-like floating structure is carried out in the ANSYS plug-in of Solidworks software. It mainly includes two aspects: pressure and deformation. The floating structure is divided into four immersion gradients: 10, 20, 30, and 38 cm. When it is immersed in water, the dynamic state of the floating structure is simulated. The deformation simulation results are shown in Figure 6b–e and the pressure results are shown in Figure 6f–i.

The magnitude of the force and deformation in Figure 6 is represented by a color gradient. Blue indicates the smallest value, and red reflects the largest value. Through the pressure analysis of the floatable structure, as the volume of the floatable structure immersed in water becomes increasingly larger, its overall deformation diminishes. The largest deformation occurs in the first stage at the bottom of the floating structure. The most serious deformation in the fourth stage is at the corners of the floating structure. In the remaining stages, there is almost no deformation. It shows that the floating structure basically has no effect on the reliability of the overall structure.

As the entire floating structure sinks deeper, the pressure area becomes larger and the overall pressure stronger. However, the color at the bottom of the T-like arch structure remains blue, indicating that the bottom structure is stable.

Combined with the static analysis of this entire process, it can be verified whether or not the floating structure can takeoff and land smoothly. After the UAV lands on the water surface, the surface area of the floating structure that touches the water surface becomes increasingly larger, and the surface tension increases. It helps the UAV to land on the water surface more smoothly. When the UAV flies from the water, the surface area of the water that the floating structure touches is diminishes. It helps to reduce the adsorption of van der Waals forces, and the UAV can leave the water more smoothly.

**Figure 7:**
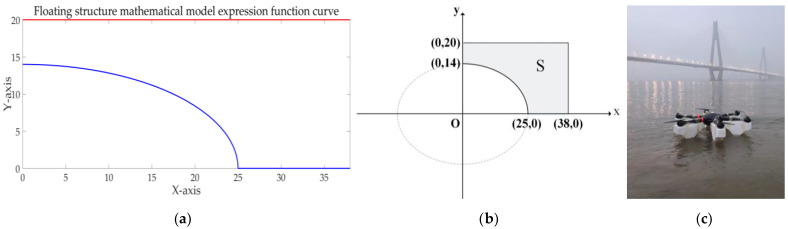
Mathematical model of floating structure and physical map: (**a**) function curve determined in MATLAB; (**b**) mathematical model; and (**c**) image of the floating UAV.

According to the change of the depth of the sinking volume, the process can be transformed into a mathematical model, as shown in Figure 7a,b. Finally, the overall physical map of the floating UAV is shown in Figure 7c.

### 2.2. Water Quality Monitoring System

This section describes the construction of a water quality monitoring system around the three main indicators that affect fishery water quality: dissolved oxygen, ammonia nitrogen, and pH. The principle of the electrode-type water quality sensor and the hardware and software aspects of the water quality detection system are elaborated in detail.

#### 2.2.1. Sensor Array

**Figure 8:**
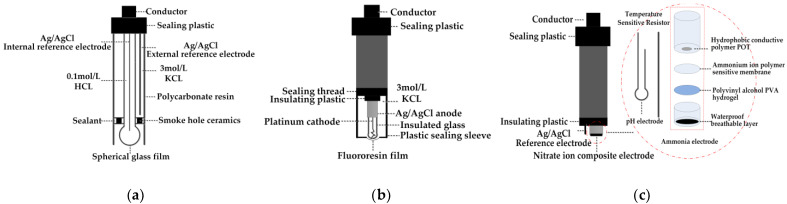
Structure diagram of three parameter sensors: (**a**) pH composite electrode; (**b**) dissolved oxygen composite electrode; and (**c**) ammonia nitrogen composite electrode.

The electrochemical water quality sensor selected for this study is an electrode-type water quality sensor. Its rapidity, high selectivity, superiority, corrosion resistance, commercial availability, and low power consumption [36] make it an excellent choice for fishery water quality detection. The pH composite electrode is shown in Figure 8a, and the specific principle is provided in other reports [37,38]. The structure of the dissolved oxygen sensor probe is shown in Figure 8b, and the specific principle is described in [39]. The schematic diagram of the ammonia-nitrogen-content water-quality-sensor motor is shown in Figure 8c, and the specific principle is given in [40].

**Figure 9:**
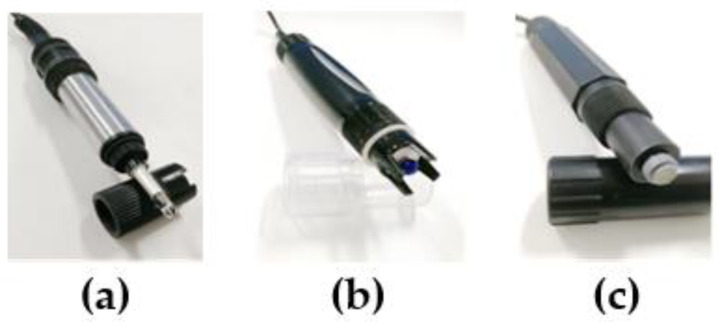
Electrode-type water quality sensor probe: (**a**) pH probe; (**b**) dissolved oxygen probe; (**c**) and ammonia nitrogen probe.

This paper selected the electrode probes of PH-300, DOB-300, ANB-300 models manufactured by BHZY (Beijing BOHAIZHIYUAN Technology CO., LTD., Beijing, China). A photo of the electrode-type water quality sensor is shown in Figure 9 and the parameters are listed in Table 3.

#### 2.2.2. Hardware Design

**Figure 10:**
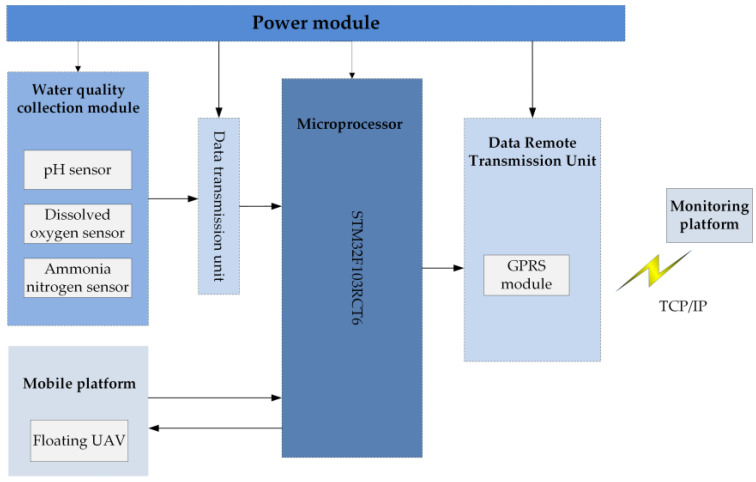
Block diagram of water quality monitoring hardware module.

According to the water quality monitoring requirements of fishery breeding, the system designed uses an air–water integrated floating UAV and fixes the sensor below the UAV to perform water quality monitoring tasks. The water quality monitoring system mainly includes a power conversion module, water quality collection module, minimum system module, data remote transmission unit, and mobile platform. The block diagram of the water quality monitoring hardware module is shown in Figure 10.

**Figure 11:**
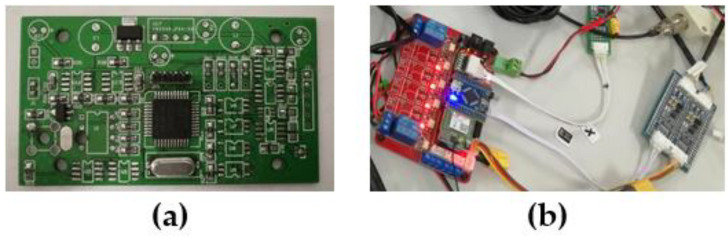
Data transmission processing unit. (**a**) The physical diagram of the hardware; (**b**) the physical diagram of the data transmission processing unit.

A photo showing the hardware design of the entire water quality detection sensor is shown in Figure 11.

#### 2.2.3. Software Design

**Figure 12:**
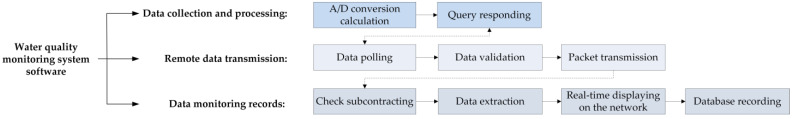
The overall design content of the water quality monitoring system software.

The purpose of the software design of the water quality monitoring system is to combine hardware to form a complete IoT monitoring system. The software part includes data collection and processing, remote data transmission, and data monitoring records. The specific software design content is shown in Figure 12.

**Figure 13:**
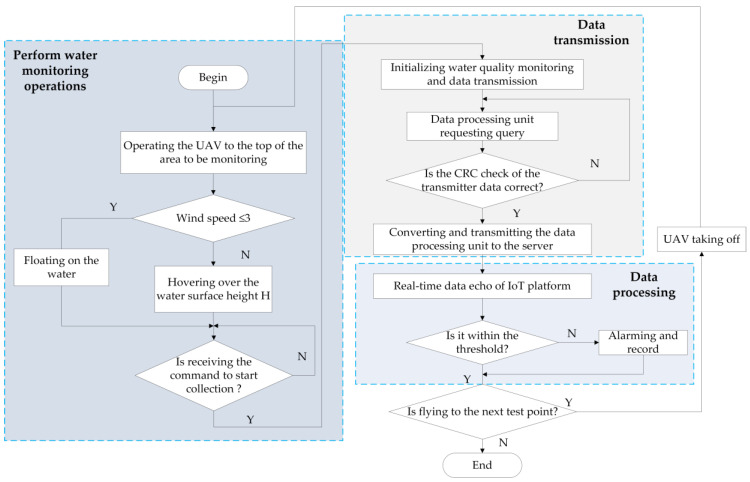
Flow chart of data collection using a UAV.

Data collection corresponds to the perception layer of the Internet of Things and is a key link in the system to obtain data. The flow chart of data collection using UAV is shown in Figure 13.

The pH, dissolved oxygen, and ammonia nitrogen content collected by the water quality sensor carried by the UAV are analog quantities, which need to be converted into digital quantities for data transmission. The conversion calculation formula follows:

(15) $$D_{out}=(U_{sensor}/U_{ref})\times (1/AD_{ratio})$$

**Figure 14:**
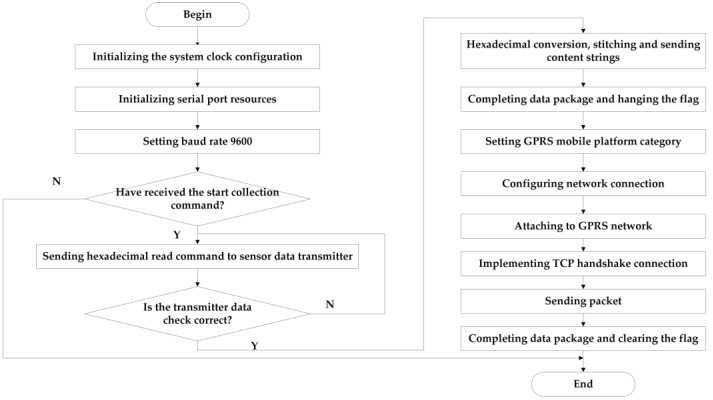
Remote data transmission flowchart.

For remote data transmission, the STM32 main controller controls the SIM800C module. It packages the water quality data of the local query response to form a message segment, and use the TCP protocol for remote transmission. The remote data transmission flowchart is shown in Figure 14.

The data monitoring record is completed by the application layer of the Internet of Things system. The design described in this report is based on the development of the Tlink Industrial Internet of Things cloud platform, which has a short development cycle and simple operation interface.

### 2.3. Water Quality Prediction 

#### 2.3.1. Water Quality Data Acquisition

**Figure 15:**
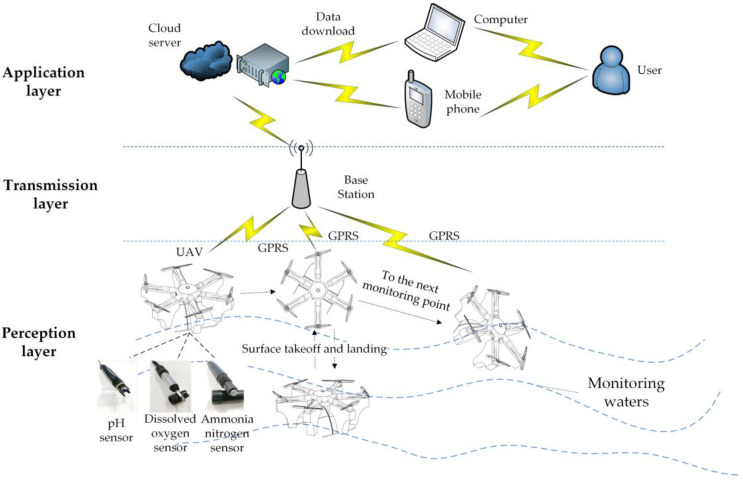
Schematic diagram of UAV water quality collection and transmission.

The data required for prediction and evaluation comes from the online monitoring of the floating UAV, which is a data transmission scheme based on the Internet of Things. The overall water quality data collection, prediction, and evaluation methods are shown in Figure 15.

#### 2.3.2. Water Quality Data Prediction Model Based on Time Series

There are three basic models of time series: autoregressive (*AR*), moving average (*MA*), and autoregressive moving average (*ARMA*) [41]. This paper assumes the *ARMA* sequence.

The p-order autoregressive process *AR*(*p*) is shown in Formula (16).

(16) $$X_{t}=c+\varphi_{1}X_{t-1}+\cdot \cdot \cdot +\varphi_{p}X_{t-p}+a_{t}$$

The q-order moving average model *MA*(*q*) is shown in Formula (17).

(17) $$X_{t}=a_{t}+\theta_{1}a_{t-1}+\cdot \cdot \cdot +\theta_{q}a_{t-q}$$

The *ARMA* model is composed of the *AR* and *MA* models, and denoted as *ARMA*(*p*, *q*):

(18) $$y_{t}=c+\varphi_{1}X_{t-1}+\cdot \cdot \cdot +\varphi_{p}X_{t-p}+a_{t}+\theta_{1}a_{t-1}+\cdot \cdot \cdot +\theta_{q}a_{t-q}$$

The *ARMA* model is based on the assumption that the data series is linear and stationary. In fact, the time series encountered often have three characteristics: trend, seasonality, and nonstationarity. An important feature of the actual time series is the existence of trend and nonlinear components; that is, the time series Y(t) is generally considered to have the following form:

(19) Y(t)=X(t)+S(t)+C(t)

Among them, X(t) is a trend component, S(t) is a seasonal component, and C(t) is a stochastic component. The *ARMA* model simulates the random component C(t). For the seasonal component S(t) (if it exists), it is usually adjusted by seasonality. For the trend component X(t), the d-order difference method is usually used to remove the trend. After making it stable, it is then fitted with the *ARMA*(*p*, *q*) model, which is the *ARIMA*(*p*, *d*, *q*) model.

In fact, *ARIMA* is a combination of the *AR* and *MA* models. The model first differentiates nonstationary time series, and then uses the *ARMA*(*p*, *q*) model for modeling.

The *ARIMA* model refers to a model established by transforming a nonstationary time series into a stationary time series, followed by regressing the dependent variable only on its lag value, the present value, and lag value of the random error term. The basic concept of the *ARIMA* model is to treat the data sequence formed by the prediction object over time as a random sequence. A certain mathematical model is then used to approximate this sequence. Once the model is identified, it can predict future values from the past and present values of the time series.

**Figure 16:**
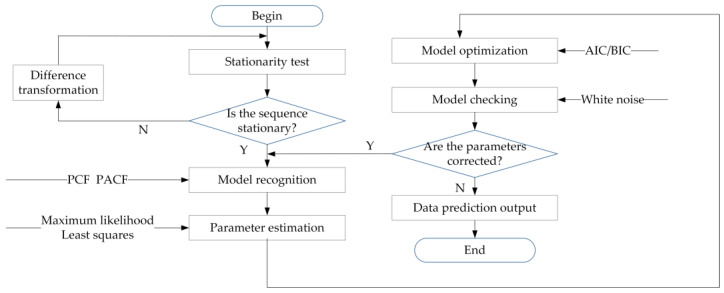
Modeling flowchart based on time series analysis and prediction.

The time series modeling steps that use the *ARIMA*(*p*, *d*, *q*) model as the research object mainly include five steps. The specific modeling flow chart is shown in Figure 16.

The first step is to check the stationarity of the original sequence. The standard method of testing is the unit root test. If the sequence does not meet the stationarity condition, mathematical methods such as difference transformation or logarithmic difference transformation can be used to make it meet the stationarity condition.

The second step is to calculate some statistics that can describe the characteristics of the sequence. For example, autocorrelation (ACF) coefficients and partial autocorrelation (PACF) coefficients are used to determine the order p and q of the *ARMA*(*p*, *q*) model. According to certain criteria, such as AIC criteria or BIC criteria, the parameters of the model are determined.

The third step is to estimate the unknown parameters of the model. The significance of the parameter and the rationality of the model are tested by the T statistic of the parameter.

The fourth step is to perform diagnostic analysis, which checks whether the residual sequence of the fitted value and the actual value of the model form a white noise sequence.

The fifth step is to perform sequence prediction analysis.

### 2.4. Water Quality Evaluation Indicators

The water quality evaluation standard adopted in this paper is based on the Fisheries Water Quality Standard GB11067–89 and the index factors pH, DO, and NH4N−+. The standard ranges of the three indicators are shown in Table 4. Combined with the single-factor evaluation index table in Table 5 “Surface Water Environmental Quality Standard GB3828-2002”, a comprehensive design of quantitative evaluation index Table 6 suitable for this paper is designed.

**Figure 17:**
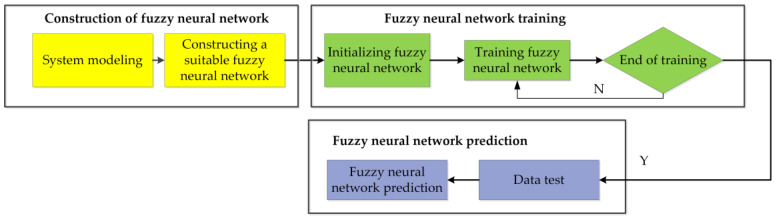
The flow chart of fuzzy neural network water quality evaluation.

In order to further compare the water quality of different regions, the T-S fuzzy neural network [42] is used to evaluate the fishery water quality. The flow chart of fuzzy neural network water quality evaluation is shown in Figure 17.

## 3. Results and Discussion

This paper carries out the work according to the hierarchical idea of field test–field experiment–algorithm simulation verification.

### 3.1. Laboratory Test

The laboratory test mainly verifies whether the floating UAV can carry electrochemical sensors for water quality monitoring.

#### 3.1.1. Water Quality Monitoring System Test

**Figure 18:**
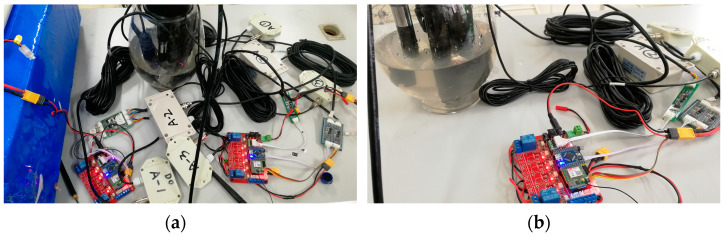
Sensor array monitoring experiment of the water quality monitoring system: (**a**) two sets of water quality monitoring hardware and (**b**) a single water quality data processing unit.

First, in order to compare and verify the reliability of the sensor, a comparative test is carried out. Two sets of water quality sensors calibrated with standard solutions are monitored and compared in liquids with unknown parameters. The built-up water quality sensor array and data processing unit are shown in Figure 18.

The comparison results of the two sets of sensors are shown in Table 7. It can be seen from the table that the performance indicators of the water quality sensor after being calibrated by the standard solution tend to be consistent. The water quality monitoring IoT platform can transmit data with the hardware of the water quality monitoring system through GPRS. The data can be saved on the server’s database for subsequent download and analysis. The water quality monitoring system can provide visual real-time monitoring.

#### 3.1.2. Floating UAV Structure Test

**Figure 19:**
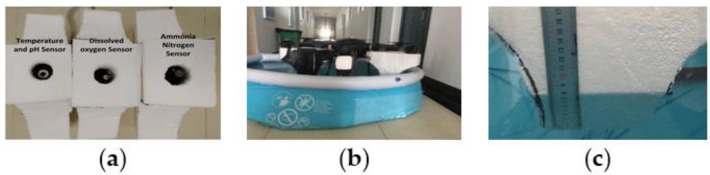
Floating structure test diagram of a floating UAV: (**a**) sensor installation diagram; (**b**) inflatable pool test; and (**c**) sinking depth of the floating structure.

Next, the floating UAV structure is tested. The structure test of the floating UAV was completed in an inflatable pool with a radius of 183 cm and a height of 51 cm. The test results are shown in Figure 19.

In a stationary situation, the total mass of the UAV is 8 kg, and the sinking height of the floating structure is about 8 cm. When the UAV is immersed on the upper edge of the arched structure, it can bear a mass of 29 kg.

### 3.2. Field Experiment

#### 3.2.1. UAV Performance Experiment 

The field experiment is more complicated than the field test, and the experiment is mainly completed in Huangjia Lake.

In order to test the stability and resistance to wind disturbance of the floating water quality monitoring UAV, a flight test was conducted outdoors. A description of the experiment follows.

**Figure 20:**
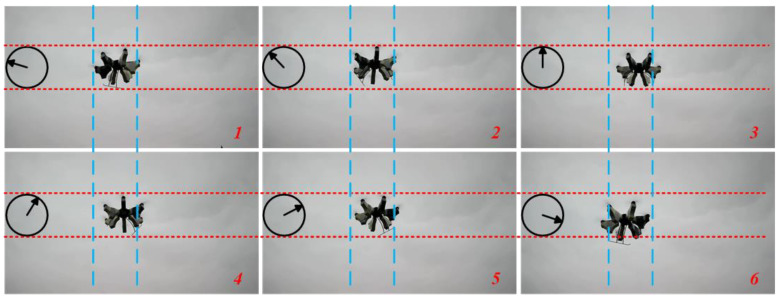
Floating water quality monitoring UAV-fixed-point rotation and yaw flight test.

This experiment uses a fixed altitude mode and yaw flight. The camera posture is kept constant, and the nose is yaw 180° for testing. In Figure 20, the position where the blue and red lines are superimposed on the screen remains unchanged. It can be intuitively observed that the UAV’s fixed-point yaw is relatively stable. When flying at a fixed point at high altitude, the position of the floating UAV changes but not by much. The wind disturbance during the normal flight of the UAV has little effect on the overall performance. Therefore, the floating water quality monitoring UAV has stable flight performance and can be used for field flight and water quality monitoring.

#### 3.2.2. Floating UAV Water Quality Monitoring

**Figure 21:**
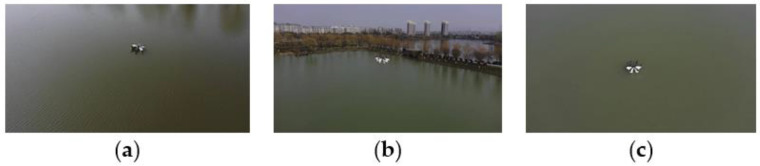
The field experiment in Huangjia Lake: (**a**) takeoff on the water surface; (**b**) the cross-regional monitoring experiment of UAV takeoff and landing; and (**c**) UAV landing experiment.

The collection experiment carried out by the floating water quality monitoring UAV in Huangjia Lake is divided into three aspects, namely, takeoff on the water surface, flying across the water surface, and floating on the water surface. The corresponding experimental results are shown in Figure 21.

When the UAV takes off and leaves the water, as the throttle increases, the motor speed increases. The buoyancy decreases, the lift increases, and the UAV gradually leaves the water surface. During the near-surface flight, it remains stable. After a short longitudinal movement, the roll, yaw, and pitch state can be quickly restored, and the overall process is very smooth.

During the landing process of the UAV, the flight near the water surface is also stable. The posture changes little, and after landing, it can quickly stabilize on the wavy water surface. Its instantaneous maximum sinking depth is about three-fifths of the entire floating structure, which is less than the upper arched edge of the floating structure. Therefore, it is safe to land on the water.

The cross-regional water takeoff and landing can also be completed smoothly, and the entire process can be very fast.

When the UAV is floating on the water, the water quality sensor array hidden in the floating structure comes into contact with the water of the measured area. The electrochemical water quality sensor is artificially triggered to start collecting data, and the data monitoring of the water quality sensor is turned off before takeoff. The data collected during the floating process can be smoothly displayed and saved in real time.

**Figure 22:**
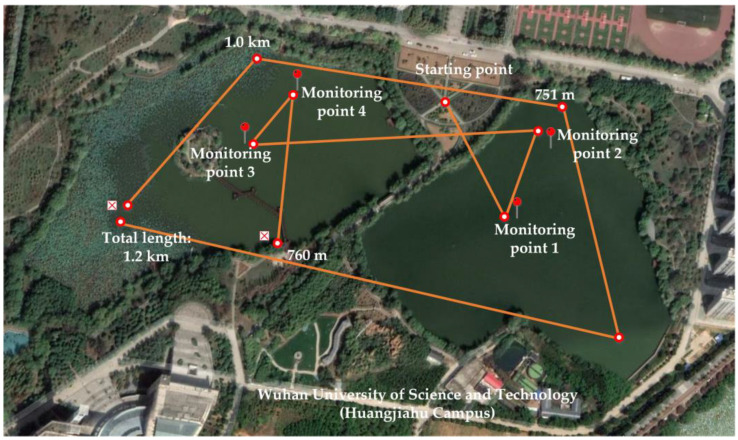
UAV route map for the entire water quality monitoring process.

The entire test process stroke is shown in Figure 22.

The details of the monitoring flight process are shown in Table 8.

It seen from the table, the entire floating water quality monitoring UAV can cover a wide range and can perform tasks for an extended time. Taking 19.80 V as the safe voltage calculation, it can theoretically perform 10 monitoring takeoff and landing tasks. Taking 30 min for one takeoff and landing floating monitoring task, the task can be continuously monitored and executed for 5 h. If it is pure floating monitoring, it can be performed for 28 h. This experiment fully demonstrates that the floating water quality monitoring UAV can give full play to its maneuverability and flexibility. It can overcome the endurance problems of conventional UAVs in completing monitoring tasks.

### 3.3. Time Series Model Prediction Experiment

The water conditions of fish ponds are closely related to weather changes. Those who do aquaculture, one very important daily task is to closely observe changes in the weather. The weather in summer is volatile. It is often accompanied by severe weather such as heavy rain, thunderstorm, continuous rain, low pressure, and sultry heat, which have a great impact on the water environment of the breeding pond. Therefore, the prediction experiment in this paper is divided into two groups: sunny and rainy.

After downloading water quality monitoring data from the cloud, the data monitored in different regions are fused in a mean weight:

(20) $$pH=\frac{\sum_{i=1}^{n}pH_{i}}{n}$$

(21) $$DO=\frac{\sum_{i=1}^{n}DO_{i}}{n}$$

(22) NH4N−+=∑i=1n[NH4N−+]in

#### 3.3.1. Sunny Experiment

**Figure 23:**
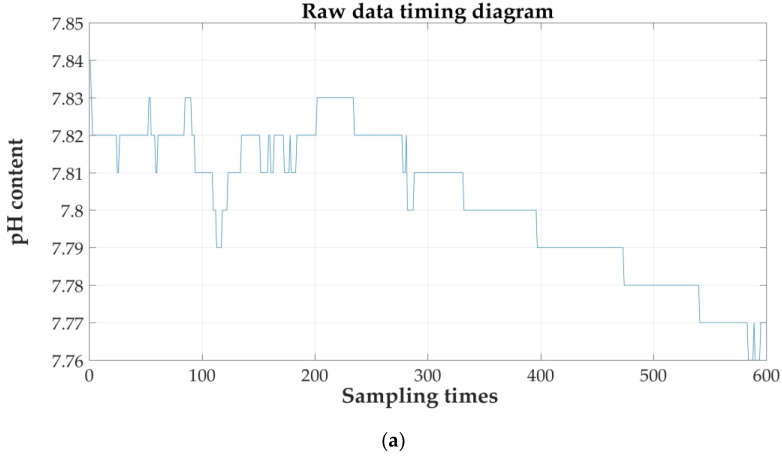

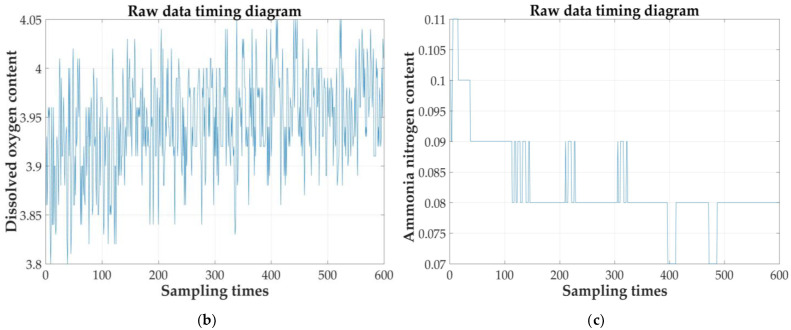
Raw data timing diagram: (**a**) pH; (**b**) DO; and (**c**) NH4N−+.

Five hundred ninety-nine sets of data in a selected continuous time period during a sunny day were imported into MATLAB for time series analysis and prediction. The raw sequence of the three sets of parameters is shown in Figure 23.

Figure 23 shows that the dissolved oxygen data have obvious sudden changes, which are considered as interference items. Next, the prediction experiment of dissolved oxygen will be explained in detail

- Prediction of dissolved oxygen content:
  1. Stationarity test.

Smoothing the dissolved oxygen data, the following processed sequence is used as a sample sequence for time series analysis.

**Figure 24:**
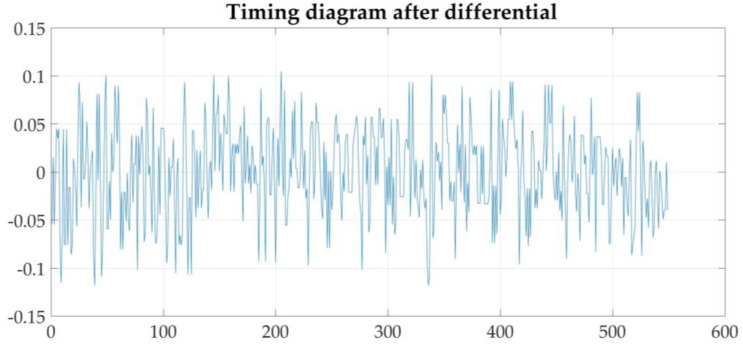
Sequence diagram of dissolved oxygen after stability treatment.

Figure 24 shows that the sequence after the first-order difference is stable near 0. The ADF unit root method is then used to verify the stationarity of the data. The return value of the data H after the first-order difference is 1. The first-order difference sequence is stable, and the significance level is 0.001—far less than 0.05. Therefore, the first-order difference sequence is stable with high probability and can be used for time series modeling.

 2. Periodic inspection.

**Figure 25:**
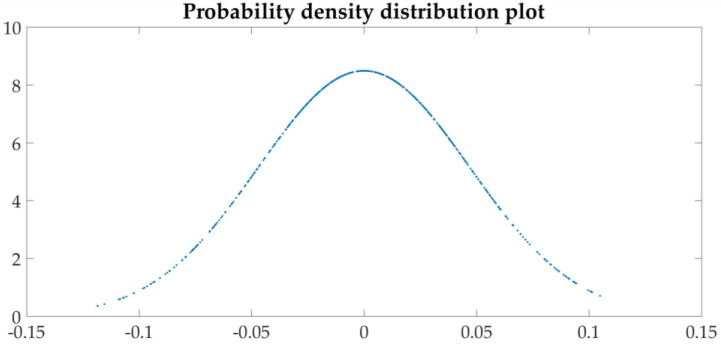
Probability density distribution.

The probability density distribution diagram of periodic data is concave, while the random data is upper convex. The probability distribution diagram of the sequence after the first-order difference of the three sets of parameters is shown in Figure 25.

The shape of the probability density distribution graph is convex (bell-shaped), which means that the time series does not have periodicity.

 3. Normality test.

**Figure 26:**
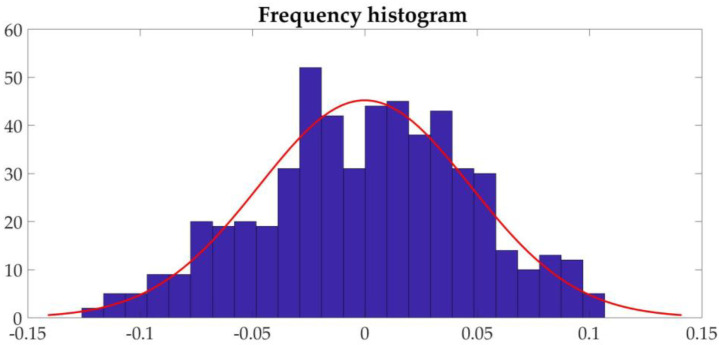
Frequency distribution histogram.

Commonly used time series models are generally based on white noise with normal distribution characteristics. Therefore, it is necessary to check whether the time series data exhibit normal characteristics. The normality test can be judged through the frequency distribution histogram. The frequency distribution histogram of the sequence after the first-order difference of the three sets of parameters is shown in Figure 26.

In the figure, the red line is obtained by normal curve fitting to the data after the first-order difference. It can be judged that the sequence satisfies the normal distribution.

 4. Model identification and order determination.

**Figure 27:**
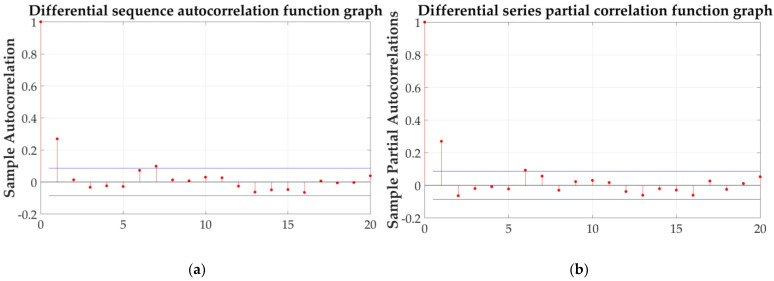
(**a**) Autocorrelation function and (**b**) partial correlation function.

By testing the autocorrelation coefficient and partial correlation coefficient, the model can be identified by its closure and tailing properties. The autocorrelation function after the first-order difference is shown in Figure 27a, and the partial correlation function is shown in Figure 27b.

Figure 27 shows that the autocorrelation function and partial correlation function of the first-order difference sequence are tailing.

According to the definition of the AIC criterion, the best fit order is obtained when the calculation result of AIC is the smallest. At this time, the pH value model is ARIMA (5,0,5), the dissolved oxygen model is ARIMA(3,0,2), and the ammonia nitrogen content model is ARIMA(5,0,5).

 5. Model checking.

**Figure 28:**
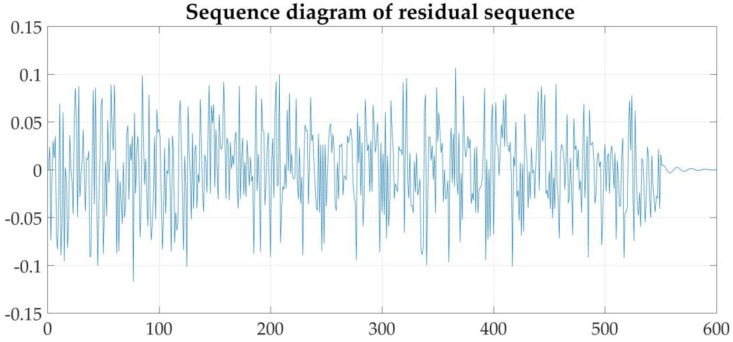
Time series diagram of the fitted residual sequence.

In order to ensure the reliability of the model built, the residual sequence of the model needs to be tested. The time sequence diagram of the fitted residual sequence is shown in Figure 28. It can be seen from the figure that the residual sequence oscillates positive and negative around 0.

**Figure 29:**
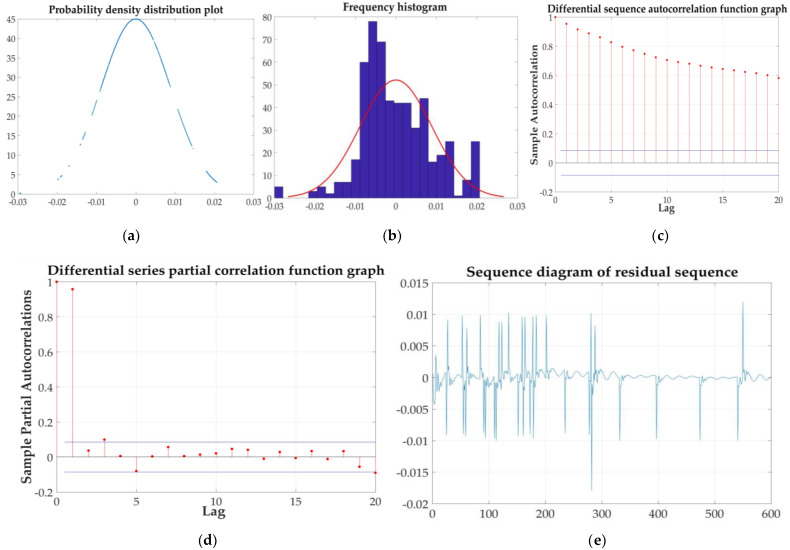
Five steps: (**a**) probability density distribution; (**b**) frequency distribution histogram; (**c**) autocorrelation function; (**d**) partial correlation function; and (**e**) fitted residual sequence.

- Prediction of pH content:

The five steps before pH value prediction: stationarity test, periodic inspection, normality test, model identification and order determination, and model checking are shown as Figure 29.

**Figure 30:**
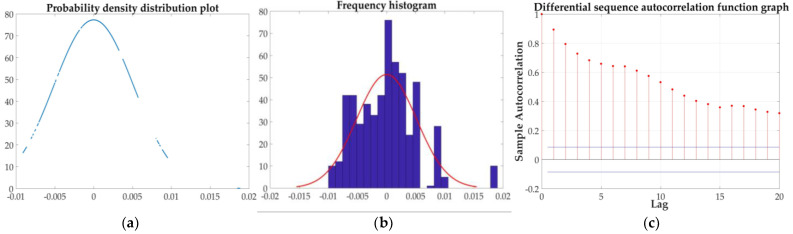

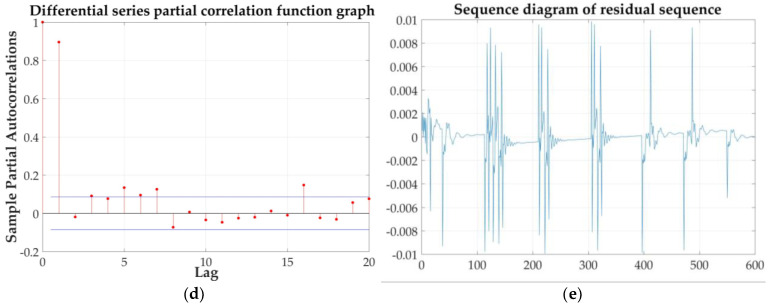
Five steps: (**a**) probability density distribution; (**b**) frequency distribution histogram; (**c**) autocorrelation function; (**d**) partial correlation function; and (**e**) fitted residual sequence.

- Prediction of ammonia nitrogen content:

Similarly, the five steps before the prediction of the ammonia nitrogen value are shown in Figure 30.

**Figure 31:**
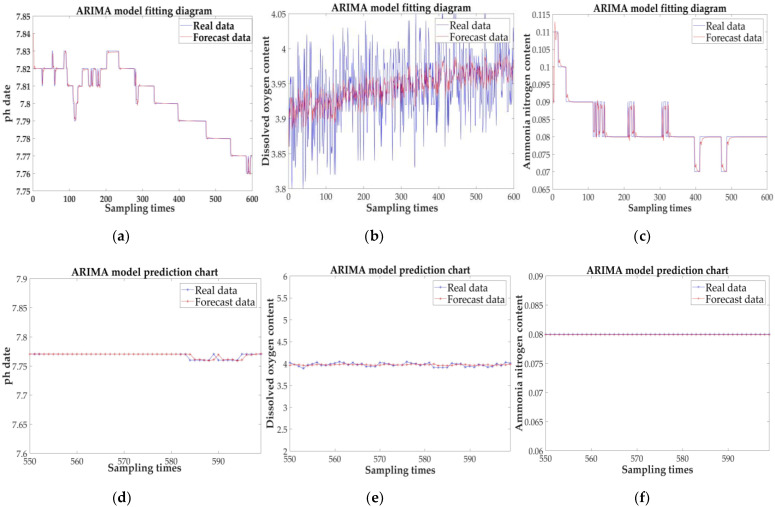
Fitting graph and prediction graph of the three parameters: (**a**) fitting effect diagram under the pH; (**b**) fitting effect diagram under the DO; (**c**) fitting effect diagram under the NH4N−+; (**d**) predicted results for pH; (**e**) predicted results for DO; and (**f**) predicted results for NH4N−+.

Finally, through the steps described above, the established ARIMA model is used to complete the prediction. As shown in Figure 31, the fitting graph and prediction graph of the three parameters show that the predicted value is basically close to the real value. The maximum error between the predicted data and the real data of 50 groups is shown in Table 9.

From the figure and table above, it is not difficult to conclude that the three index predictions have good fitting effects; from the perspective of the maximum relative error, the accuracy rates reached 99%, 98%, and 99%. This also reflects from the side that dissolved oxygen changes in water generally fluctuate greatly, while pH and ammonia nitrogen content are stable. In general, the prediction results are relatively accurate and can be used as a basis for predicting fishery water quality.

#### 3.3.2. Rainy Experiment

The experimental data are made with 100 sets of data collected on a rainy day. The data are divided into two parts. The first part consists of 90 sets of data for time series modeling. The second part is 10 sets of data as experimental data for model prediction. Through stationary, periodic, and normality tests, together with model identification, order selection, and a model test, the prediction is completed.

**Figure 32:**
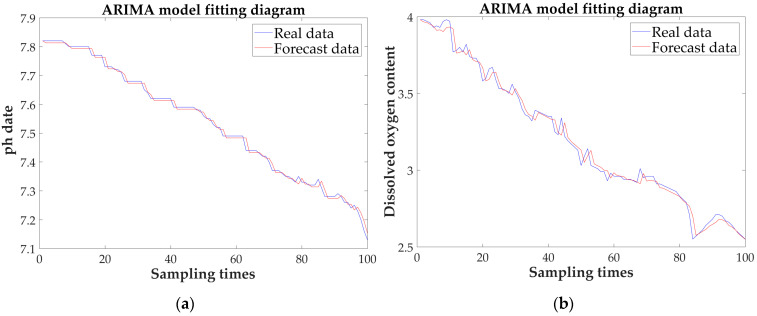

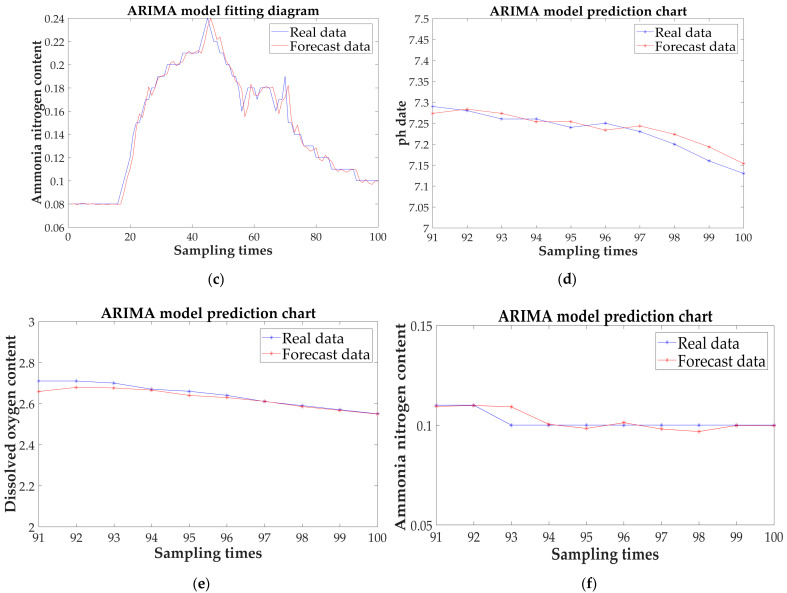
Fitting graph and prediction graph of the three parameters: (**a**) fitting effect diagram under the pH; (**b**) fitting effect diagram under the DO; (**c**) fitting effect diagram under the NH4N−+; (**d**) predicted results for pH; (**e**) predicted results for DO; and (**f**) predicted results for NH4N−+.

Figure 32 shows that the predictions of the three parameters have a good fitting effect. In Table 10, the prediction accuracy rates of the three parameters reach 92%, 98%, and 91%, respectively. Compared with the result of the sunny day experiment, the deviation is large. Moreover, the overall values for pH and dissolved oxygen decrease, and the ammonia nitrogen content increases.

The monitoring data show that the pH is about 7.8 on a sunny day and drops to 7.1 on a rainy day. It is because the ability of algae to absorb carbon dioxide during photosynthesis decreases, which increases the carbon dioxide content in the water body, and finally the pH value decreases. The dissolved oxygen content is about 4 during sunny days, while it is reduced to 2.55 during rainy days. Because of the rainy weather, low temperature, and weak light intensity, the photosynthesis of phytoplankton in the water body declines rapidly. The oxygen supply capacity of the water body is poor, and the dissolved oxygen in the water decreases. The silt at the bottom of the pond undergoes an anaerobic reaction due to long-term hypoxia. A large number of substances are produced, such as methane, hydrogen sulfide, ammonia nitrogen, nitrite, and other substances that are harmful and toxic to fish growth. Therefore, the ammonia nitrogen content even rises from about 0.08 to 0.24, when compared with sunny days.

Therefore, an aerator can be used flexibly in the rainy season. Together with the use of reagents that inhibit the growth and reproduction of harmful microorganisms at the bottom of the pool, it can degrade various algae poisons, ammonia nitrogen, nitrite, and other toxic substances. In addition, aeration replenishes beneficial bacteria in time to maintain the microecological balance of the water body.

### 3.4. Water Quality Evaluation Quantification

**Figure 33:**
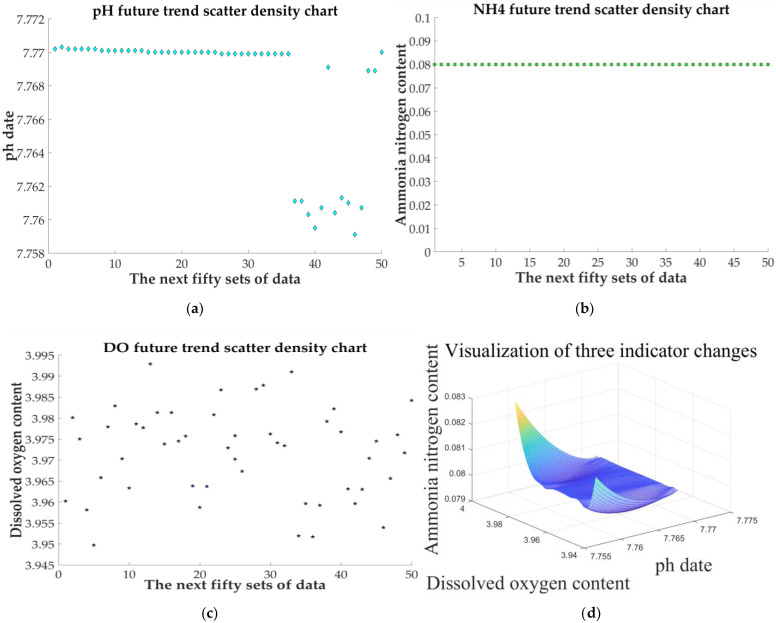
The scatter plots and the overall visualization of the three sets of data changes: (**a**) pH; (**b**) NH4N−+; (**c**) DO; and (**d**) visualization of three indicator changes.

The 50 sets of data in the sunny day predicted by time series model are used for quantitative evaluation and analysis. The scatter plots of the three sets of data changes are shown in Figure 33a–c.

From the perspective of time change, the overall water quality of the monitored lake is alkaline. The data are relatively concentrated (Figure 33a), which meets the “excellent” indicator in Table 6. The ammonia nitrogen content is relatively stable (Figure 33b), and most of the data conform to the Table 6 “excellent” indicator. In Figure 33c, the reason for the lack of concentration of dissolved oxygen in a short period of time may be that the dissolved oxygen content of water is not saturated, and most of the data are at the lower edge of the “excellent” indicator given in Table 6.

To comprehensively evaluate the impact of the three indicators, 50 sets of predicted data are displayed in three-dimensional visualization, as shown in Figure 33d.

It is not difficult to see from Figure 33d that the changes are mainly concentrated in the dissolved oxygen and pH, which can also be seen from Figure 33a,b. The visual view constructed by the three water quality indicators, however, is close to one surface as a whole, indicating that the overall situation is relatively stable.

**Figure 34:**
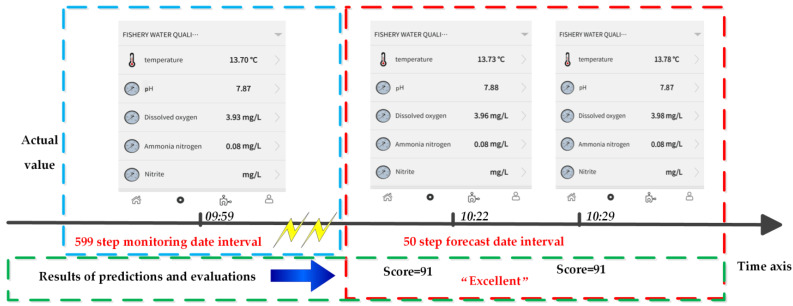
Actual value of water quality monitoring at a certain moment.

Taking the actual monitoring data corresponding to a certain moment in the 50 step prediction interval as an example, combining with Table 6 and substituting Formula (23) for quantitative calculation, the total score is 91, and the evaluation level is “excellent”. The results is shown in Figure 34.

(23) $$Score=pH_{Grade}\times 0.4+DO_{Grade}\times 0.3+NH_{4}^{+}-N_{Grade}\times 0.3$$

These results are consistent with the prediction and evaluation data results. It shows that the future water quality of the monitoring area is generally good and suitable for a fishery, which is also consistent with the actual situation.

In order to further test the differences in water quality in different regions, four monitoring points were selected for evaluation, as shown in Figure 22. Ten sets of data are selected for each monitoring point, and the result of the T-S fuzzy neural network evaluation is shown below.

**Figure 35:**
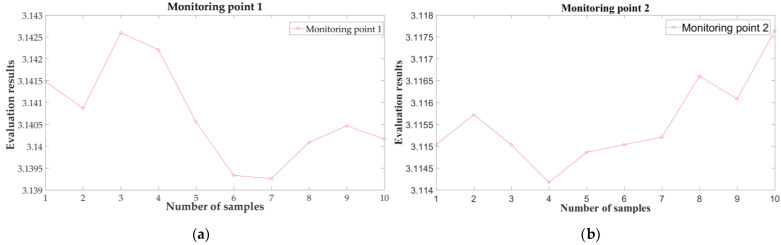

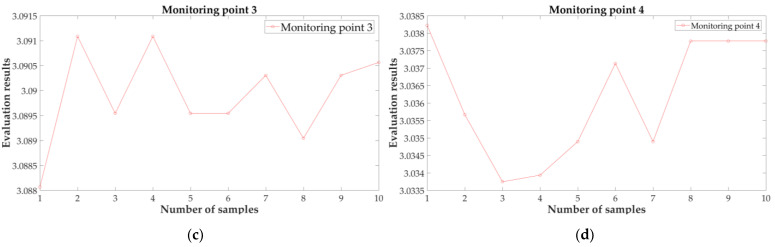
The water quality in different regions: (**a**) monitoring point 1; (**b**) monitoring point 2; (**c**) monitoring point 3; and (**d**) monitoring point 4.

From Table 4 and Figure 35, it can be seen that the water quality of the four regions is “good” or above, which meets the fishery water quality requirements.

## 4. Conclusions

This paper introduces in detail the solutions for long-term water quality monitoring and evaluation of future water quality trends using UAV. First, a complete water quality monitoring software and hardware experimental platform for floating UAVs is calculated, including the water quality monitoring system and the design of floating UAV platforms. Combined with the method of time series comprehensive analysis, the ARIMA model is then established to realize the prediction of water quality. The evaluation rule table is established to conduct a comprehensive evaluation of the predicted data. Finally, the experiment and result analysis show that the water quality monitoring and prediction evaluation system based on the floating UAV can successfully complete the water quality monitoring task of landing–floating–takeoff on the water surface. It provides a data visualization display of the Internet of Things platform, and the data can be stored and analyzed smoothly. The ARIMA model established by time series analysis can accurately realize water quality prediction. Combined with the evaluation rules, the predicted water quality status can be comprehensively analyzed. The future scope of the work aims at studying the optimization of the floating structure to achieve the best effect.

## Figures and Tables

**Table 1:** Water quality prediction methods.

Classification				Use	Limitation
**Nonmechanical methods**	Gray model prediction [16,17,18]			Make medium and long-term forecasts	Large deviation
	Time series analysis [19,20,21,22]	Random time series method	Markov	Manage data that are difficult to express as a function of time	Large amount of data support
			Box–Jenkins		
		Deterministic time series	Time series smoothing	The original data changes have a certain law	Cannot handle data that are difficult to express as a function of time
			Trend extrapolation		
			Seasonal variation forecast		
	Artificial neural network prediction [23,24,25]	Long Short-Term Memory (deep learning method)		Improve unstable time series with more fixed components.	High requirements for development time, data volume, and calculation cost
		Back Propagation neural network		Nonlinear time series data	
**Mechanism methods**	Water quality numerical model [26,27,28]				Need extensive hydrological data modeling

**Table 2:** Water quality evaluation method.

Classification Method	Evaluation Method
Stage	Review evaluation
	Present situation evaluation
	Prejudge evaluation
Use	Agricultural irrigation
	Industrial production
	Domestic drinking
Index number relationship	Single-factor evaluation
	Multi-factor evaluation
Type of water body	Surface water quality evaluation
	Groundwater quality evaluation
Method	Fuzzy comprehensive evaluation method [29,30,31]
	Artificial neural network evaluation method

**Table 3:** Parameters of three electrode probes.

Parameter Index	pH Probe	Dissolved Oxygen Probe	Ammonia Nitrogen Probe
Measuring range	0–14 pH	0–20 mg/L	0–10 mg/L
Resolution	0.01 pH, 0.1 °C	0.01 mg/L, 0.1 °C	0.01 mg/L, 0.1 °C
Precision	±0.02 pH, ±0.2 °C	±0.5% FS, ±0.3 °C	±1% FS, ±0.3 °C
Output load	<300 Ω	<300 Ω	<300 Ω
Working voltage	DC12V	DC12V	DC12V

**Table 4:** Fishery water index ranges corresponding to the three index factors studied in this paper.

Index Factor	Index Range
pH	6.5–8.5 (freshwater)
DO	≥3 mg/L
NH4 ± N	≤2 mg/L

**Table 5:** Surface Water Environmental Quality Standard GB3828-2002.

Index Factor	Class I	Class II	Class III	Class IV	Class V
pH	7.0–8.0	6.0–7.0/8.0–9.0	5.0–6.0/9.0–10.0	4.0–5.0/10.0–11.0	0–4/11–14
DO	8	6–8	4–6	2–4	0–2
NH4N−+	0–0.2	0.2–0.5	0.5–1.0	1.0–1.5	2.0

**Table 6:** Water quantitative evaluation index.

Quantify Grade/Range/Score	pH	DO	NH4N−+	Network Output Range (3.0–3.3)
Excellent/(90–100)/100	6–8	4–8	≤0.2	3.0–3.1
Good/(70–90)/70	5–6 or 8–9	2–4 or 8–10	0.2–0.3	3.1–3.2
Poor/(<70)/40	<5 or >9	<2 or >10	>0.3	3.2–3.3
Weight	0.4	0.3	0.3	

**Table 7:** The monitoring results of two sets of water quality sensors connected to the IoT platform.

	Water Quality Monitoring Experiment	Water Quality Monitoring Comparison Experiment
pH	6.68 pH	6.68 pH
Dissolved oxygen	4.75 mg/L	4.45 mg/L
Ammonia nitrogen content	0.15 mg/L	0.14 mg/L
Temperature	12.5 °C	12.4 °C

**Table 8:** Monitoring flight process indicators.

Index	Value	Index	Value	Index	Value
Number of monitoring points	4	The distance between monitoring points 1 and 2	102 m	Monitoring region area	200,000 m^2^
Number of takeoffs	4	The distance between monitoring points 2 and 3	208 m	Task execution time	157 min
Number of landings	4	The distance between monitoring points 3 and 4	70 m	Starting point voltage	25.25 V
Total number of takeoffs and landings	5	Total horizontal route	760 m	End point voltage	22.60 V

**Table 9:** Analysis of the maximum error in the prediction results of pH value, dissolved oxygen content, and ammonia nitrogen content in the sunny experiment.

Sequence	Real Data	Predicted Data	Absolute Error	Relative Error
pH	7.77	7.7595	0.0105	0.135%
DO	3.89	3.9581	−0.0681	1.751%
NH4 ± N	0.08	0.079875	0.000125	0.156%

**Table 10:** Analysis of the maximum error in the prediction results of pH value, dissolved oxygen content, and ammonia nitrogen content in the rainy experiment.

Sequence	Real Data	Predicted Data	Absolute Error	Relative Error
pH	7.77	7.16	0.61	7.851%
DO	2.71	2.659	0.051	1.882%
NH4 ± N	0.1	0.1092	−0.0092	9.2%

## Data Availability

Data available on request without restrictions, e.g., privacy or ethical. The data presented in this study are available on request from the corresponding author. Some data are not publicly available due to real-time monitoring during the experiment.
